# Impact of Progerin Expression on Adipogenesis in Hutchinson—Gilford Progeria Skin-Derived Precursor Cells

**DOI:** 10.3390/cells10071598

**Published:** 2021-06-25

**Authors:** Farah Najdi, Peter Krüger, Karima Djabali

**Affiliations:** Epigenetics of Aging, Department of Dermatology and Allergy, TUM School of Medicine, Technical University of Munich (TUM), 85748 Garching, Germany; Farah.najdi@tum.de (F.N.); Peter.krueger@tum.de (P.K.)

**Keywords:** progerin, senescence, adipocyte, skin-derived precursor cells, adipogenesis

## Abstract

Hutchinson–Gilford progeria syndrome (HGPS) is a segmental premature aging disease caused by a mutation in *LMNA*. The mutation generates a truncated and farnesylated form of prelamin A, called progerin. Affected individuals develop several features of normal aging, including lipodystrophy caused by the loss of general subcutaneous fat. To determine whether premature cellular senescence is responsible for the altered adipogenesis in patients with HGPS, we evaluated the differentiation of HGPS skin-derived precursor stem cells (SKPs) into adipocytes. The SKPs were isolated from primary human HGPS and normal fibroblast cultures, with senescence of 5 and 30%. We observed that the presence of high numbers of senescent cells reduced SKPs’ adipogenic differentiation potential. Treatment with baricitinib, a JAK–STAT inhibitor, ameliorated the ability of HGPS SKPs to differentiate into adipocytes. Our findings suggest that the development of lipodystrophy in patients with HGPS may be associated with an increased rate of cellular senescence and chronic inflammation.

## 1. Introduction

Hutchinson–Gilford progeria syndrome (HGPS, OMIM 176670) is a rare genetic disease that causes premature aging in children. Currently, approximately 135 cases of HGPS are documented worldwide [1]. Affected individuals die at an average age of 14.7 years owing to coronary failure or stroke [2]. Patients with HGPS exhibit several symptoms of normal aging, such as alopecia, atherosclerosis, loss of joint mobility, and severe lipodystrophy [3,4]. In most cases, the disease results from a de novo mutation in the *LMNA*, which encodes the A-type nuclear lamin. The C–T transition in exon 11 at nucleotide 1824 of the *LMNA* creates a cryptic splice site, resulting in the formation of a truncated form of prelamin A, named progerin [3,5]. Owing to this mutation, progerin undergoes an incomplete post-translational processing, and remains permanently farnesylated and irreversibly anchored to the nuclear membrane, which disrupts the lamina meshwork and function [6,7]. The functional alterations include uncoupling of the heterochromatin from the nuclear envelope, formation of nuclear blebs, and an increase in DNA damage with a reduction in the DNA damage repair response [7,8,9,10,11].

Lipodystrophy is a rare disorder characterized by the total or partial loss of the adipose tissue [12]. Adipose tissue is an endocrine and highly active metabolic organ that plays an important role in energy homeostasis [13]. It has also been implicated in the regulation of several functions, including blood pressure, reproductive functions, angiogenesis, and immune response [14,15,16]. In healthy individuals, fat accumulates in white adipose tissue (WAT) in the form of triglycerides [17]. The fat storage capacity of WAT tissues is impaired in patients with lipodystrophy, resulting in the accumulation of fat in visceral deposits [18]. Abnormal lipid accumulation and storage in these visceral deposits induces lipotoxicity, which may lead to increased cardiovascular risk, hepatic steatosis, and insulin resistance [19].

Subcutaneous fat loss in children with HGPS can be detected during the first year after birth [20,21]. Fat loss is initiated in the limbs, spreads to the thorax and neurocranium, and subsequently becomes evident in the face, with the disappearance of the buccal fat pads [21,22]. The thinning of the skin due to the loss of subcutaneous fat makes the blood vessels more prominent, particularly those on the face and scalp [23]. Additionally, patients only gain limited weight per year, starting at 24 months of age [3]. The patients fail to thrive and lose body fat despite adequate caloric intake [2].

Mutations in *LMNA* are known to affect lipid metabolism and adipocyte differentiation in patients [24,25]. In vivo studies on the *Lmna^G609G/G609G^* mouse model, which harbors the primary mutation in *LMNA* implicated in HGPS, revealed the loss of body weight and subcutaneous fat in the mice due to progerin expression [26]. The prevention of progerin accumulation by therapeutic intervention reversed this phenotype [27]. In fact, progerin expression in mice showed depletion in the adipose tissue with aging along with increased senescence and inflammation [28]. Progerin expression also reduced the potential of adipocyte differentiation in both induced pluripotent stem cells (iPSCs) and human mesenchymal stem cells (hMSCs) derived from patients with HGPS. The differentiated adipocytes showed alterations in their lipid storage capacity [29,30].

To gain additional insights into the molecular mechanisms underlying the development of lipodystrophy in patients with HGPS, we established an ex vivo cellular model to dissect adipogenesis in HGPS. Adult stem cells isolated from patients with HGPS were needed. However, the acquisition of biopsies was not an option, owing to the rarity and juvenile mortality of the patients. To circumvent this problem, we opted for a method previously established by us to isolate skin-derived precursor cells (SKPs) from pre-established human primary fibroblast cultures [31]. SKPs are multipotent stem cells found in the dermis [32]. They have recently gained attention due to their advantages and potential applications in therapeutics [33,34,35]. Importantly, they are present during adulthood and express stem cell markers [32,36,37]. The isolation and expansion of SKPs from normal dermal fibroblast cultures using the low-pH stress method was described recently [38]. In this study, we demonstrate that the isolation of SKPs from HGPS primary fibroblast cultures and their differentiation into adipocytes was possible. Our findings indicate the prominent role of senescence in inhibiting adipogenesis. We also showed that the use of baricitinib, a selective JAK 1/2 inhibitor [39], could enhance adipogenesis in SKPs derived from HGPS fibroblast cultures by delaying senescence.

## 2. Materials and Methods

### 2.1. Cell Culture

The human primary dermal fibroblast cell lines, GMO5565 (3-year-old male), GMO1651 (13-year-old female) and GMO5567A (12-year-old male) were all obtained from the Coriell Institute for Medical Research (Camden, NJ, USA). HGPS cell lines were obtained from the Progeria Research Foundation Cell and Tissue Bank. The following HGPS cell lines were used: HGADFN003 (2-year-old male) and HGADFN127 (3-year-old female). The 3T3-L1 (ATCC^®^ CL-173™) cell line was purchased from ATCC (Manassas, VA, USA). Fibroblast cell lines were cultured as monocultures in DMEM (Thermo Fisher—Gibco, Waltham, MA, USA, D6429) supplemented with 15% fetal bovine serum (FBS) (Thermo Fisher—Gibco, 10270106), 1% L-glutamine (Thermo Fisher—Gibco, 25030081), 1% penicillin/streptomycin (Thermo Fisher—Gibco, 1514022) and 0.5% gentamycin (Thermo Fisher—Gibco, 15710049). The fibroblasts were subcultured and used when they were approximately 80% confluent. All cultures were performed in a cell incubator (Binder, Tuttlingen, Germany, 9140-0046) in a humidified chamber at 37 °C and 5% CO_2_.

### 2.2. Low-pH SKP Isolation and Culture

Primary fibroblast cultures (80% confluent) were collected by trypsin-EDTA (Thermo Fisher—Gibco, 25200056) and pelleted at 1200 rpm for 5 min at room temperature (RT), and washed once with phosphate-buffered saline (PBS) buffer. One million cells were resuspended in 500 μL of pH-adjusted Hank’s balanced salt solution (HBSS) buffer (Thermo Fisher—Gibco, 14175053). The pH of the HBSS buffer was adjusted to 5.7 using HCL (Merck KGaA, Darmstadt, Germany, 1.00319.2500). Cells suspended in HBSS buffer with pH 5.7 were incubated for 25 min at 37 °C and agitated every 5 min. The cell suspensions were centrifuged for 5 min at 1200 rpm at RT. Next, each pellet (containing 1 × 10^6^ cells) was suspended in 6 mL of SKP media [37] (4:1—DMEM low glucose (Thermo Fisher—Gibco, 21885025):F12 (Thermo Fisher—Gibco, 21765029), 20 ng/mL EGF (Thermo Fisher—Gibco, PHG0311), 40 ng/mL bFGF (Thermo Fisher—Gibco, PHG0026), 2% *v*/*v* B27 (Thermo Fisher—Gibco, 17504044), 0.5 μg/mL fungizone (Thermo Fisher—Gibco, 15290018), and 100 U/100 μg/mL penicillin/streptomycin) and the suspension was equally divided into two T25 non-tissue-culture-treated flasks (Fisher Scientific—Falcon, Hampton, NH, USA, 10112732). The cultures were supplemented every other day with 10 x SKP media (SKP media with 10× concentrated EGF, bFGF, and B27) diluted to a final concentration of 1× in culture media and agitated daily by pipetting up and down to prevent the adherence of the spheroids to the plastic flask.

### 2.3. Differentiation of SKPs into Adipocytes

SKPs at day 4 were collected and washed twice with PBS. In case of adherence, the spheroids were incubated in adherence media (SKP media supplemented with 5% FBS) for 24 h, then switched to adipocyte differentiation media (ADM) (DMEM supplemented with 4.5 g/L glucose (Thermo Fisher—Gibco, 21885025), 0.5 mM 3-isobutyl-1-methylxanthine (IBMX, Sigma-Aldrich, St. Louis, MO, USA, I7018, stock in absolute ethanol [VWR chemicals, Radnor, PA, USA, 20821.33]), 10 μg/mL insulin (Sigma-Aldrich, I2643, stock in 0.01 M HCL [Merck KGaA, 1.00319.2500] in Ultra-Pure water from MilliQ [MQ]), 100 µM L-Ascorbic Acid (Sigma, A8960, stock in Ultra-Pure water from MilliQ [MQ]), 1 μM dexamethasone (Sigma-Aldrich, D4902, stock in absolute ethanol), 10% FBS, 0.5 μg/mL fungizone, 50 μM indomethacin (Sigma-Aldrich, I7378, stock in 100% DMSO [Sigma-Aldrich, D2650]), and 100 U/100 μg/mL penicillin/streptomycin). For trypsinization, the spheroids were dissociated with trypsin-EDTA (Thermo Fisher—Gibco, 25200056) and then seeded onto 0.2% gelatin-coated cover slips in a 24-well plate. In the control SKP group, 8 × 10^4^ dissociated SKPs were seeded per well, whereas in the HGPS SKP group, 10^5^ dissociated SKPs were seeded per well. The cells were cultured in the ADM according to the method described above.

Baricitinib (Absource Diagnostics GmbH, Munich, Germany), 1 µM, was added to the SKP medium and ADM. The ADM was replaced every 2–3 days.

### 2.4. Differentiation of 3T3-L1 Cells

The 3T3-L1 preadipocytes were cultured at 37 °C in 5% CO_2_-enriched air in DMEM (Thermo Fisher—Gibco, D6429) supplemented with 15% FBS (Thermo Fisher—Gibco, 10270106), 1% l-glutamine (Thermo Fisher—Gibco 25030081), 1% penicillin/streptomycin (Thermo Fisher—Gibco, 1514022), and 0.5% gentamycin (Thermo Fisher—Gibco, 15710049). Cells were seeded in 6-well plates on glass cover slips at a density of 2 × 10^5^ cells per well. After confluence was reached, differentiation was induced by replacing the medium with ADM (composition described above). After 72 h, the medium was replaced with DMEM supplemented with 10% FBS, 10 μg/mL insulin, and 100 U/100 μg/mL penicillin/streptomycin (ADM II) for 4 days, with one medium change during this period. On day 7, the medium was replaced with DMEM supplemented with 10% FBS and 100 U/100 μg/mL penicillin/streptomycin (basal medium). This medium was refreshed every second day until day 14 of differentiation.

### 2.5. Senescence Associated Beta-Galactosidase (SA-β-Gal)

Senescence was assessed using the protocol from Dimri et al. [40]. The HGPS and control fibroblasts were washed with PBS and fixed for 5 min in 0.2% glutaraldehyde (Sigma-Aldrich, G5882)/2% formaldehyde (Sigma-Aldrich, 104003). Cells were then washed twice with PBS and incubated overnight at 37 °C (in the absence of CO_2_) in SA-β-Gal staining solution (5 mM potassium ferricyanide (Merck KGaA, 104973), 5 mM potassium ferrocyanide (Sigma-Aldrich, P9387), 2 mM MgCl_2_ (Sigma-Aldrich, M-1028), 150 mM NaCl (Sigma-Aldrich, 310166), 0.5 mg/mL 5-bromo4-chloro-3-indolyl P3-D-galactoside (X-gal) (Sigma-Aldrich, 3117073001), and 40 mM citrate/sodium phosphate buffer (pH 6.0). Blue-stained cells were considered as positive, and on average, 1000 cells were counted from each sample.

### 2.6. Oil Red O (ORO) Staining

The adipocytes obtained in vitro were fixed with 4% paraformaldehyde (PFA) (Merck KGaA, 104005) for 30 min. Cells were incubated in 60% isopropanol for 5 min, and then in ORO solution for 5 min. The ORO working solution was prepared by mixing 15 mL of the stock solution which contained Oil Red O powder (Sigma-Aldrich, O0625) in 99% isopropanol with 10 mL demineralized water, filtered using a Whatman filter paper. The slides were rinsed two times with tap water and were screened under a microscope.

### 2.7. Bodipy Staining

Differentiated adipocytes from the control and HGPS SKP groups were treated with 2 µM Bodipy (Invitrogen, Waltham, MA, USA, D3922) diluted in PBS for 15 min at 37 °C. The slides were washed twice with PBS and fixed in 4% PFA for 20 min. The cells were then washed three times with PBS and counterstained with DAPI Vectashield mounting medium (Vector Laboratories, Burlingame, CA, USA, VEC-H-1200).

For immunofluorescence imaging of the Bodipy-stained cells, Bodipy was added with a secondary antibody in blocking buffer at a concentration of 2 µM for 1 h.

### 2.8. Immunocytochemistry

Cells were grown on glass cover slips and fixed either with ice-cold methanol or 2% PFA. Cells were either fixed for 10 min at −20 °C in methanol or in PFA at RT and then washed with PBS. Following PFA fixation, the cells were permeabilized with 0.2% Triton X-100 in PBS for 10 min at RT and washed two times with PBS.

Mouse anti-p16INK4A (Sigma-Aldrich, P0968, 1:250, overnight), mouse anti-p21 (Invitrogen, MA5-14949, 1:250, overnight), mouse anti-IL-8 (CXCL8) (Invitrogen, M801 1:400, 3 h), rabbit anti-progerin (overnight) [41], mouse anti-PPARγ (E8) (Santa Cruz Biotechnology, Dallas, TX, USA, sc-7273, 1:100, 2 h), rabbit anti-FABP4 (Sigma-Aldrich, HPA002188, 1:100, 2 h), and mouse anti-Lamin A (Sigma-Aldrich, L1293, 1:2000, 2 h) antibodies were used as primary antibodies. The secondary antibodies used were affinity-purified Alexa Fluor^®^ 555 or 488 conjugated anti-rabbit/mouse antibodies (Life Technologies, Carlsbad, CA, USA, A21206 anti-rabbit-488, A21202 anti-mouse-488, A31572 anti-rabbit-555, and A31570 anti-mouse-555, 1:500/1000).

Following fixation, the cells were blocked for 30 min with 10% FBS in PBS. The primary antibodies were diluted in the blocking buffer at the concentrations and incubation periods mentioned for each antibody. Antibodies incubated for a few hours were at RT, whereas antibodies incubated overnight were placed at 4 °C. Next, the cells were rinsed three times with PBS, before the secondary antibodies diluted in the blocking buffer were added for 1 h at RT. Following incubation with the secondary antibody, cells were washed 3 times with PBS. Next, cells were stained with DAPI Vectashield mounting medium (Vector Laboratories, VEC-H-1200). Images were acquired using an Axio Imager D2 fluorescence microscope (AxioCam MRm, Carl Zeiss, Oberkochen, Germany).

### 2.9. Image Analysis

The images were analyzed, the brightness/contrast was adjusted with Fiji [42], and the images were imported to Adobe Photoshop CC 2017 for illustration.

### 2.10. Statistical Evaluation and Graphics

The results are presented as mean ± SD and were compared using the Student’s *t*-test or two-way ANOVA. The symbols used to indicate statistical significance include: ns, not significant, *p* > 0.05, * *p* ≤ 0.05, ** *p* ≤ 0.01, and *** *p* ≤ 0.001. The calculations were performed and the graphs were constructed using Prism version 6.01 (GraphPad, San Diego, CA, USA).

## 3. Results

### 3.1. Characterization of HGPS SKPs

**Figure 1:**
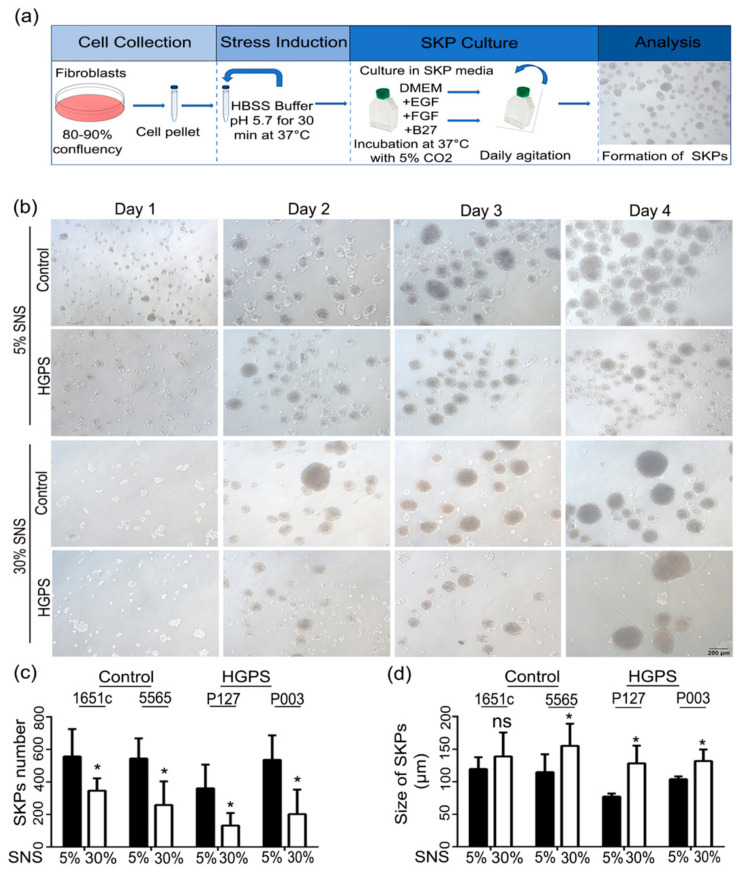
Isolation of SKPs from control and HGPS fibroblasts. (**a**) Panel showing the protocol for SKP isolation. Briefly, fibroblasts were pelleted and treated with HBSS buffer (pH 5.7) for 30 min at 37 °C. Cells were cultured in SKP media containing DMEM low glucose, EGF, FGF, and B27. The flasks were agitated daily, and the spheroids were harvested at day 4 for analysis. (**b**) SKP formation from both control (GMO1651c, GMO5565) and HGPS (HGADFN127, HGADFN003) fibroblasts with 5 and 30% senescence (SNS). (**c**,**d**) Quantification of the number and the diameter of the spheroids from control and HGPS fibroblast cultures with 5 and 30% SNS at day 4. Values are presented as mean ± SD (*n* = 3), not significant (ns), * *p* < 0.05, (**c**,**d**) unpaired *t*-test. HBSS: Hank’s Balanced Salt Solution, DMEM: Dulbecco′s modified Eagle medium, EGF: epidermal growth factor, FGF: fibroblast growth factor, SKPs: skin-derived precursor cells, SNS: senescence.

Previous studies on SKPs showed that SKP spheroids could be generated from primary normal fibroblast cultures using a low-pH stress procedure [38]. To determine whether HGPS primary fibroblast cultures also contained SKPs, we used the same procedure [38], as outlined in Figure 1a.

Senescence and the senescence-associated secretory phenotype (SASP) have been implicated in stem cell depletion [43]. In addition, cultures of fibroblasts obtained from patients with HGPS are known to exhibit premature senescence [44]. Spheroids were obtained from the different cell strains starting with fibroblast cultures at low (<5% senescence (SNS)) and high (~30% SNS) senescence indexes (Figure 1b). The SKP yield was considerably higher in preparation derived from young (early passages) control and HGPS fibroblast cultures, and had an average size of 117 µm for control SKPs and 90 µm for HGPS SKPs (Figure 1c,d). The SKP spheroids derived from old fibroblast cultures (30% SNS) were fewer and appeared to be larger, with an average size of 147 µm for control SKPs and 130 µm for HGPS SKPs (Figure 1c,d). Collectively, our findings indicate that SKP spheroid isolation and expansion from HGPS fibroblast cultures can be achieved using the low-pH SKP protocol, as reported previously for normal fibroblast cultures [38]. However, the SKP spheroid yield was inversely correlated with the number of senescent cells present in the fibroblast cultures of both cell types.

### 3.2. Differentiation of SKP Spheroids into Adipocytes

**Figure 2:**
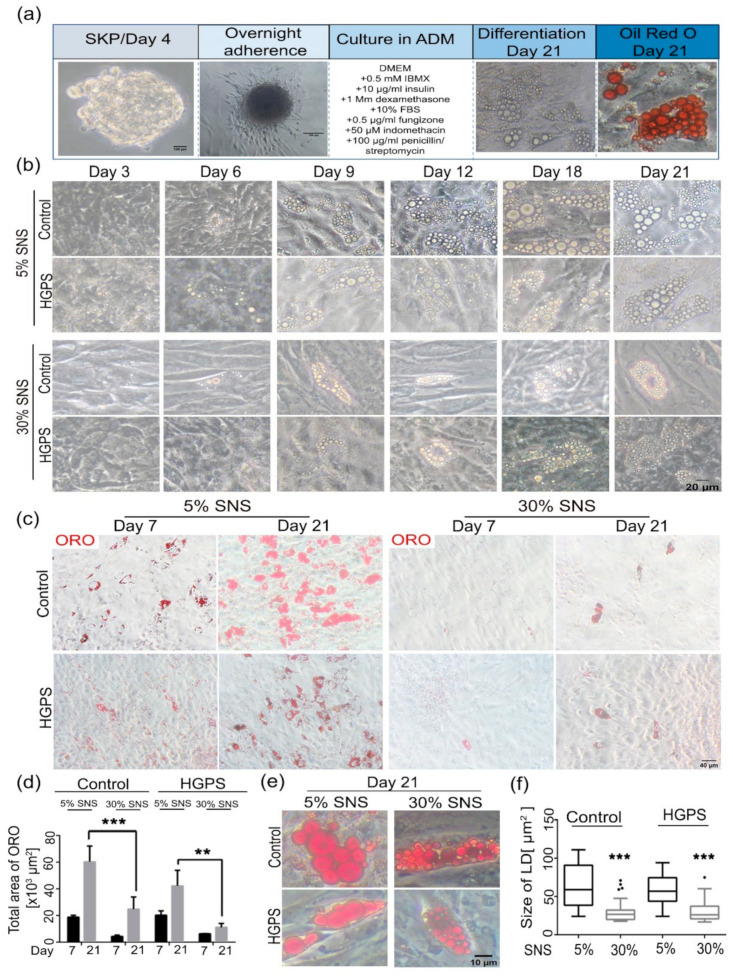
Differentiation of control and HGPS SKPs into adipocytes. (**a**) Panel showing the protocol of SKPs adherence and differentiation. SKPs were collected at day 4 and allowed to adhere overnight in the presence of SKP adherence media. Next, the media was replaced with ADM supplemented with insulin, IBMX, and dexamethasone. Differentiation occurred for 21 days, and the differentiated adipocytes were stained with Oil Red O (ORO) at day 21. (**b**) Bright-field imaging of the differentiation of control and HGPS SKPs with 5 and 30% SNS into adipocytes at different time points. (**c**) ORO staining for differentiated control and HGPS SKPs originating from 5- and 30%-senescence fibroblast cultures at day 7 and 21 of adipogenesis. (**d**) Quantification of total ORO-stained area using Image J. (**e**,**f**) Quantification of the size of lipid droplets in control and HGPS groups with 5 and 30% SNS at day 21 of differentiation. (**d**) Values are presented as mean ± SD (*n* = 3). (**f**) In the Box and Whisker plot, the horizontal line crossing the box is the median, the bottom and top of the box are the lower and upper quartiles, the whiskers are the minimum and maximum values, and the dots represent the outliers, ** *p* < 0.01, *** *p* < 0.001, (**d**) two-way ANOVA with Tukey’s multiple comparisons test, (**f**) unpaired *t*-test. FBS: fetal bovine serum, IBMX: isobutylmethylxanthine.

Stem cells derived from patients with HGPS exhibit impaired adipogenesis potential [30]. To determine whether this defect is attributed to the presence of a high number of senescent cells, we induced adipocyte differentiation in young and old SKPs derived from both control and HGPS fibroblast cultures. The previously described adipocyte differentiation protocol was used [38], based on which the SKP spheroids were allowed to adhere overnight in the presence of adipocyte adherence media, and then switched to ADM for 21 days with media changes (Figure 2a). The differentiation of SKP spheroids from young (<5% SNS) and old (>30% SNS) fibroblast cultures of control and HGPS groups was observed at different time points (Figure 2b). On day 6, a few cells exhibited lipid droplets in both HGPS and control young cultures (Figure 2b, panel 5% SNS). From day 9 to 21, the number and size of the lipid droplets increased in both cell types. Notably, during adipogenesis of SKPs derived from old fibroblast cultures, lipid droplets were barely detectable on day 6 in control and HGPS (Figure 2b, panel 30% SNS). From day 9 to day 21, very few cells showed the presence of lipid droplets, and the droplets remained small in size at day 21 in both cell types (Figure 2b).

To further evaluate the differentiation potential of the SKP spheroids, lipid vesicles were labeled with ORO on day 7 and 21 of differentiation (Figure 2c) and the total area of staining was quantified using Fiji [42] (Figure 2d). The total area with an ORO signal was relatively similar in both HGPS and control adipocytes derived from young fibroblast cultures (Figure 2d). However, in adipocytes derived from SKPs of old fibroblast cultures, the lipid content was lower in both cell types (Figure 2d). The size of the lipid droplets on day 21 of differentiation was measured using Fiji. The average size of lipid droplets in adipocytes derived from old fibroblasts was smaller than that in adipocytes derived from young counterparts in both control and HGPS groups (Figure 2e,f). High magnification of the HGPS and control adipocytes showed the presence of larger lipid droplets in cells derived from young fibroblast cultures (<5% SNS) than in those derived from old fibroblast cultures (>30% SNS) (Figure 2e,f).

Collectively, these results show that HGPS SKP spheroids isolated from young fibroblast cultures can differentiate into adipocytes, which further accumulate large lipid droplets of sizes similar to that in their control counterparts on day 21.

### 3.3. Characterization of the Senescence Index of SKP Spheroids

**Figure 3:**
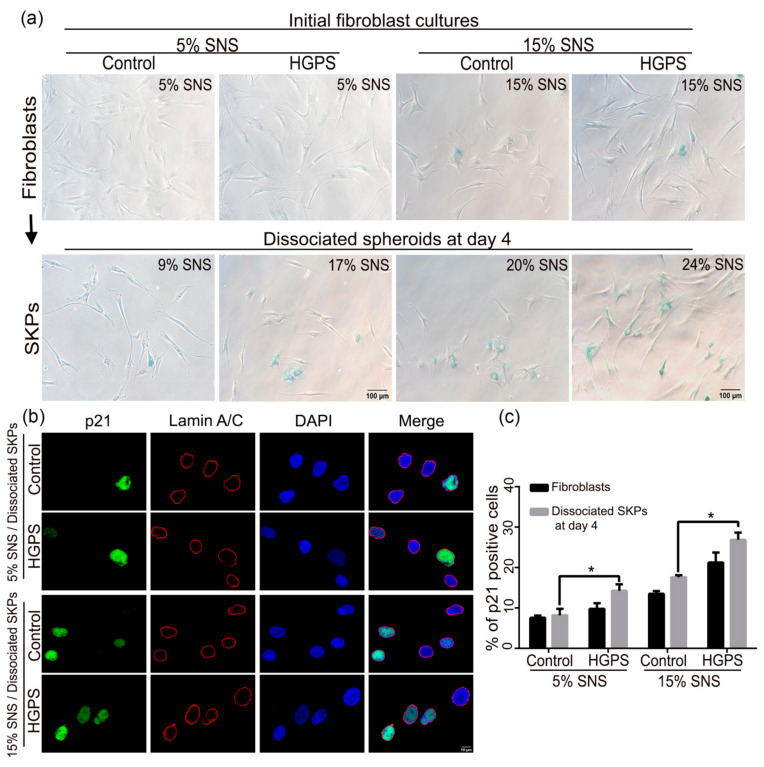
Senescence index of SKPs: SA-β-gal and p21 staining of original fibroblasts and dissociated SKPs at day 4. (**a**) SA-β-gal test performed using initial fibroblast cultures and on dissociated spheroids at day 4 for both control and HGPS groups starting from 5 and 15% SNS. (**b**) Immunofluorescence staining for p21 and lamin A/C in dissociated spheroids at day 4, from control and HGPS SKPs with 5 and 15% SNS. Cells were counterstained with DAPI. (**c**) Quantification of the percentage of p21-positive nuclei in the initial fibroblast cultures and in dissociated SKPs at day 4 in both control and HGPS groups. Values are presented as mean ± SD (*n* = 3), * *p* < 0.05, (**c**) two-way ANOVA with Tukey’s multiple comparisons test.

In Figure 1 and Figure 2, we demonstrate that the senescence index of the primary fibroblast cultures negatively affected SKP spheroid preparation and differentiation. To determine whether a proportion of cells entered senescence and further negatively affected adipogenesis during the SKP preparation, we measured the levels of senescence in SKP preparations at day 4 (Figure 3 and Figure S1).

For this, we performed SA-β-gal staining directly on SKPs from both control and HGPS cultures (Figure S1a). However, SA-β-gal staining of spheroids was not possible as the dye remained trapped within these structures (Figure S1a). To circumvent this issue, day 4 spheroids were dissociated and allowed to adhere overnight in SKP adherence media prior to SA-β-gal staining (Figure 3a). In parallel, we also performed SA-β-gal staining on the starting control and HGPS fibroblast cultures and on the corresponding dissociated spheroids (Figure 3a). When the 5% SNS control fibroblast cultures were used, the corresponding dissociated spheroids showed a 4% average increase in senescence on day 4, whereas the spheroids derived from HGPS cultures showed a 12% increase (Figure 3a). The same analysis performed on starting fibroblast cultures with 15% SNS led to an increase of 5% SNS in control dissociated SKPs and a 9% increase in the HGPS counterparts (Figure 3a).

**Figure 4:**
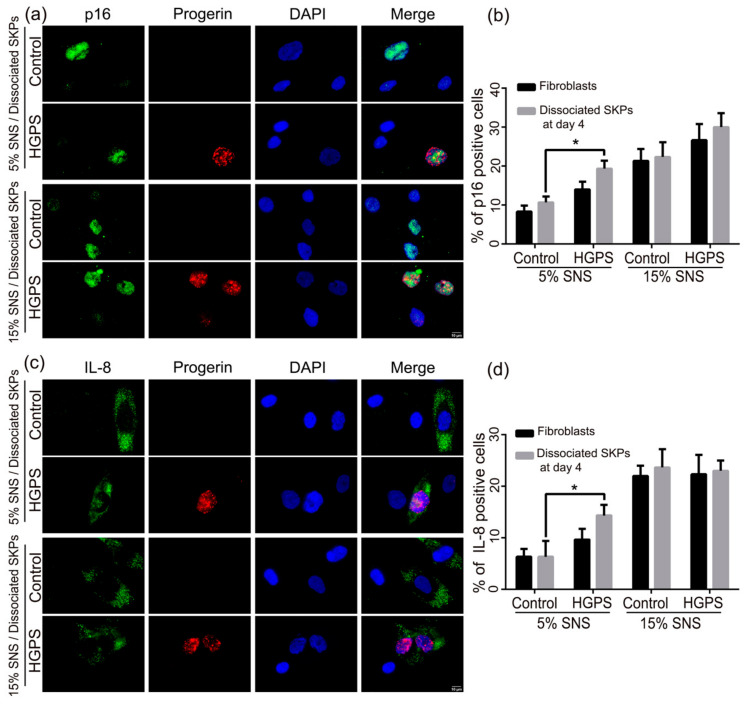
Immunofluorescence staining for p16 and IL-8 in dissociated control and HGPS SKPs at day 4. (**a**) Immunofluorescence staining for p16 and progerin at day 4 in dissociated spheroids from control and HGPS SKPs with 5 and 15% SNS. (**b**) Quantification of the percentage of p16-positive nuclei in the initial fibroblast cultures and in dissociated SKPs at day 4 from both control and HGPS cultures. (**c**) Immunofluorescence staining for IL-8 and progerin at day 4 in dissociated spheroids from control and HGPS SKPs with 5 and 15% SNS. Cells were counterstained with DAPI. (**d**) Quantification of the percentage of IL-8-positive cells at day 4 in the initial fibroblast cultures and in dissociated SKPs from both control and HGPS cultures. Values are presented as mean ± SD (*n* = 3), * *p* < 0.05, (**c**) two-way ANOVA with Tukey’s multiple comparisons test.

To validate these findings, immunofluorescence staining was performed for three additional senescence markers: p21 (Figure 3b,c and Figure S1b), p16INK4A (p16) (Figure 4a) and IL-8 (Figure 4c and Figure S2) [45,46,47]. The number of p21-positive dissociated SKPs at day 4 increased among both control and HGPS preparations derived from initial fibroblast cultures with 5% and 15% SNS (Figure 3c). The scoring of p16- and IL-8-positive SKPs indicated a similar increase in the proportion of cells expressing these markers (Figure 4). The percentage of p16- and IL-8 positive cells was higher in dissociated SKPs at day 4 than in the corresponding starting fibroblast cultures for both cell types at 5 and 15% SNS (Figure 4b,d). In addition, all senescent markers were more increased in HGPS-derived SKPs than in controls.

These findings indicate that the pH-SKP isolation protocol induces cellular stress, allowing the isolation and expansion of SKPs present in fibroblast cultures; however, this stress may render the cells more susceptible to senescence, particularly in the case of HGPS SKPs. Therefore, the increased rate of senescence in HGPS SKPs could affect their potential for differentiation into adipocytes.

### 3.4. Adipogenesis of Dissociated SKP Spheroids Derived from Young Fibroblast Cultures

**Figure 5:**
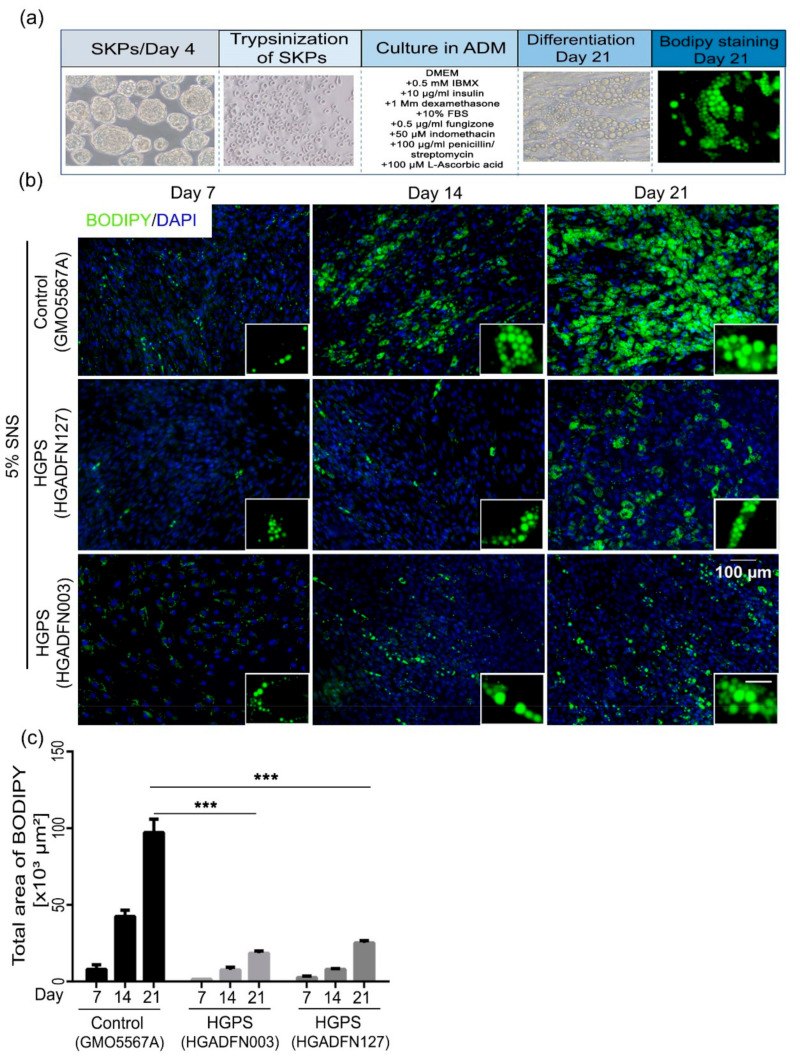
Differentiation of trypsinized SKPs into adipocytes. (**a**) Panel showing the optimized protocol for SKP trypsinization at day 4 and differentiation into adipocytes. The spheroids were trypsinized at day 4 and cultured directly in ADM. The differentiated cells were observed for 21 days and then stained with ORO and Bodipy at day 21. (**b**) Bodipy staining of lipid vesicles for control (GMO5567A) and HGPS (HGADFN127 and HGADFN003) cells, starting from 5% SNS fibroblast cultures. Staining was performed at days 7, 14, and 21 of differentiation. Cells were counterstained with DAPI. Scale bar: 100 µm; for the magnified images, scale bar: 20 µm. (**c**) Quantification of area with Bodipy signals at the three time points for both control and HGPS adipocytes. Values are presented as mean ± SD (*n* = 3), *** *p* < 0.001, (**c**) two-way ANOVA with Tukey’s multiple comparisons test.

Although the previously described differentiation protocol (Figure 2a) led to functional adipocyte differentiation, it had certain limitations. The whole spheroids adhered before differentiation. Therefore, the number of spheroids added to each well could not be monitored rigorously, rendering the quantification of the differentiation potentials of HGPS versus control spheroids difficult to assess. To optimize this protocol, SKP spheroids were first dissociated, and the SKP cell number was determined. After several trials, we determined the following seeding conditions; for HGPS, 100,000 dissociated SKPs were seeded per well (in a 24-well plate), and 80,000 dissociated SKPs were seeded for the control. Next, the SKPs were cultured in ADM for a period of 21 days (Figure 5a).

Staining with Bodipy and ORO were performed at three different time points: days 7 (early stage of differentiation), 14, and 21 (mature adipocytes) (Figure 5b and Figure S3). The total area with Bodipy staining was quantified using Fiji. SKPs isolated from control fibroblast cultures showed efficient differentiation capacity, as indicated by Bodipy and ORO staining (Figure 5b and Figure S3). HGPS spheroids derived from the two HGPS cell strains (HGADFN003 and HGADFN127) showed impaired adipocyte differentiation, as indicated by the reduced Bodipy and ORO signals compared with control counterparts (Figure 5b,c and Figure S3). At days 7, 14 and 21, the total area showing Bodipy staining was significantly smaller in differentiated HGPS adipocytes than in the control (GMO5567A) (Figure 5c). The area of Bodipy staining on day 21 of adipocyte differentiation in HGPS was four times smaller than that in control counterparts (Figure 5c).

Collectively, the adipocyte differentiation protocol, starting with the dissociation of SKPs, indicated that HGPS SKPs exhibit impaired adipogenesis compared to control SKPs. The presence of more senescent cells in HGPS SKP preparations could be attributed to the reduced adipogenic potential of HGPS SKPs.

### 3.5. Effect of Baricitinib, a Specific JAK1/2 Inhibitor, on Spheroid Formation and Adipogenesis

**Figure 6:**
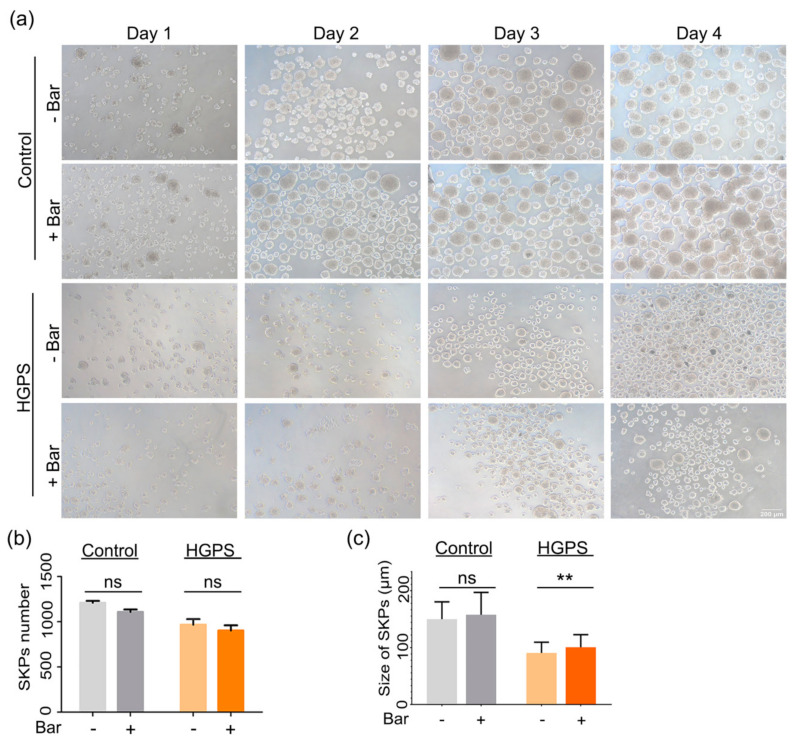
SKPs characterization after treatment with baricitinib. (**a**) SKPs isolation from control (GMO5567A) and HGPS (HGADFN127) fibroblasts after treatment with baricitinib (Bar). (**b**,**c**) Quantification of the number and average size of SKPs in control and HGPS groups with or without baricitinib treatment. Values are presented as mean ± SD (*n* = 3), not significant (ns), ** *p* < 0.01, (**b**,**c**) unpaired *t*-test.

We previously reported that HGPS fibroblasts treated with the JAK1/2 inhibitor baricitinib showed a reduction in the expression of proinflammatory markers and restoration of cellular homeostasis [44]. Accordingly, we tested whether baricitinib could also ameliorate adipogenesis in HGPS SKPs. As previously determined, 1 µM of baricitinib showed no cytotoxicity during the long-term treatment in vitro [44]. Consequently, 1 µM of baricitinib was used for treatment of control and HGPS spheroid expansion and adipocyte differentiation (Figure 6). SKP spheroid formation and expansion were unaffected by the presence of baricitinib in both the control and HGPS cultures (Figure 6a,b). The number of SKP spheroids and their average sizes were scored on day 4, and both parameters showed no obvious changes between the mock- and baricitinib-treated SKP cultures (Figure 6b,c). In control SKP cultures, the number of spheroids ranged from 1100 to 1200 per flask, whereas in the HGPS cultures, the average number ranged from 900 to 970 spheroids per flask (Figure 6b). Baricitinib treatment did not affect the size of the SKP spheroids in the control and led to an average spheroid size of approximately 150 µm on day 4 (Figure 6c). In HGPS cultures, treatment with baricitinib increased the average spheroid size (diameter) from 90 to 100 µm (Figure 6c). Collectively, these findings indicate that 1 µM baricitinib did not induce cytotoxicity or changes in SKP formation and expansion during the 4 day culture period.

**Figure 7:**
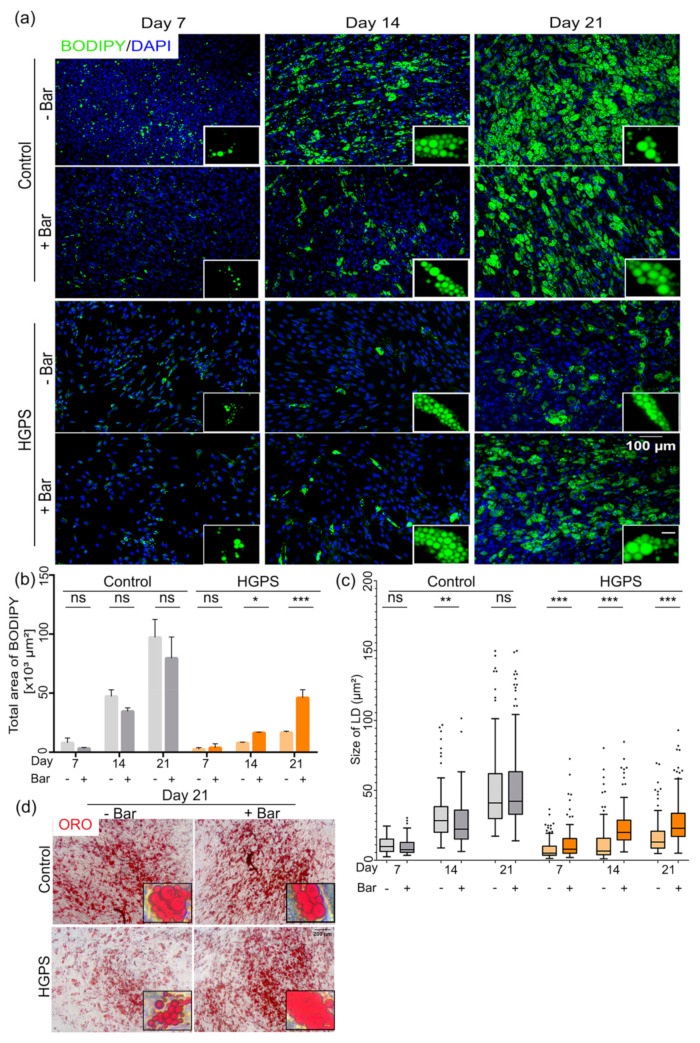
Improved differentiation of HGPS SKPs after treatment with baricitinib. (**a**) Bodipy staining of control (GMO5567A) and HGPS (HGADFN127) differentiated adipocytes after treatment with baricitinib. Scale bar: 100 µm; for the magnified images, scale bar: 20 µm. Cells were counterstained with DAPI. (**b**,**c**) Quantification of total area showing Bodipy signal and average size of lipid droplets (LD) at days 7, 14, and 21 of differentiation for both control and HGPS adipocytes. (**d**) ORO images at day 21 of differentiation for control and HGPS adipocytes. (**b**) Values are presented as mean ± SD (*n* = 3), (**c**) In the box and whisker plot, the horizontal line crossing the box is the median, the bottom and top of the box are the lower and upper quartiles, the whiskers are the minimum and maximum values, and the dots represent the outliers, not significant (ns), * *p* < 0.05, ** *p* < 0.01, *** *p* < 0.001, (**b**) two-way ANOVA with Tukey’s multiple comparisons test, (**c**) unpaired *t*-test.

To investigate whether baricitinib treatment improves the differentiation potential of the SKPs, spheroids derived from control and HGPS cultures (<5% SNS) were dissociated on day 4 and cultured in ADM with or without baricitinib (Figure 7). Adipocyte differentiation was monitored, and the samples were stained with Bodipy at days 7, 14, and 21 (Figure 7a). The total area with the Bodipy signal and the size of the lipid droplets were measured and analyzed (Figure 7b,c). Baricitinib-treated control SKPs led to similar adipocyte differentiation levels as in the mock-treated controls (Figure 7a,b). Moreover, the average size of the lipid droplets in the baricitinib-treated controls remained similar to that in mock-treated controls (Figure 7c). Baricitinib-treated HGPS SKPs showed a significant increase in adipocyte differentiation and lipid vesicle formation on day 14 (Figure 7b). Hence, on day 21 of adipocyte differentiation, the total area with Bodipy signal in baricitinib-treated HGPS cultures was 2.5-fold greater compared with mock-treated HGPS cultures (Figure 7b). Hence, the size of the lipid droplets in HGPS adipocytes were much larger in baricitinib-treated cultures (Figure 7c).

These findings indicate that baricitinib treatment improved the potential of HGPS SKPs to differentiate into adipocytes. Therefore, baricitinib could be considered to ameliorate adipogenesis in HGPS.

### 3.6. Expression of PPARγ and FABP4 in Control and HGPS Adipocytes

To characterize HGPS-derived adipocytes, we analyzed the expression profile of two well-characterized markers of adipogenesis: peroxisome proliferator-activated receptor gamma (PPARγ) and fatty-acid-binding protein 4 (FABP4) [48,49]. First, we assessed the specificity of the antibodies recognizing these two markers using 3T3-L1 preadipocytes, a well-established model for adipogenesis [50] (Figure S4).

PPARγ is an early marker of adipogenesis [51,52], and FABP4 is a marker of mature adipocytes [49]. Using the differentiation protocol outlined in Figure S4a, 3T3-L1 cells showed high efficiency for adipocyte differentiation (Figure S4b). The intensity of Bodipy expression increased from day 3 to day 14 (Figure S4b). Similar results were obtained with ORO staining (Figure S4b). Next, we scored the expression of PPARγ and FABP4 at days 3, 7, and 14 of 3T3-L1 differentiation by calculating the percentage of positive cells expressing each marker compared to the total DAPI count. PPARγ expression increased from day 3 to day 7 and remained high on day 14 (Figure S4d,e). The expression of FABP4, a marker of mature adipocytes, increased from day 3 to day 14 (Figure S4f,g). Hence, the direct counts of the FABP4-positive cells indicated 60% differentiation efficiency on day 14 (Figure S4f,g).

**Figure 8:**
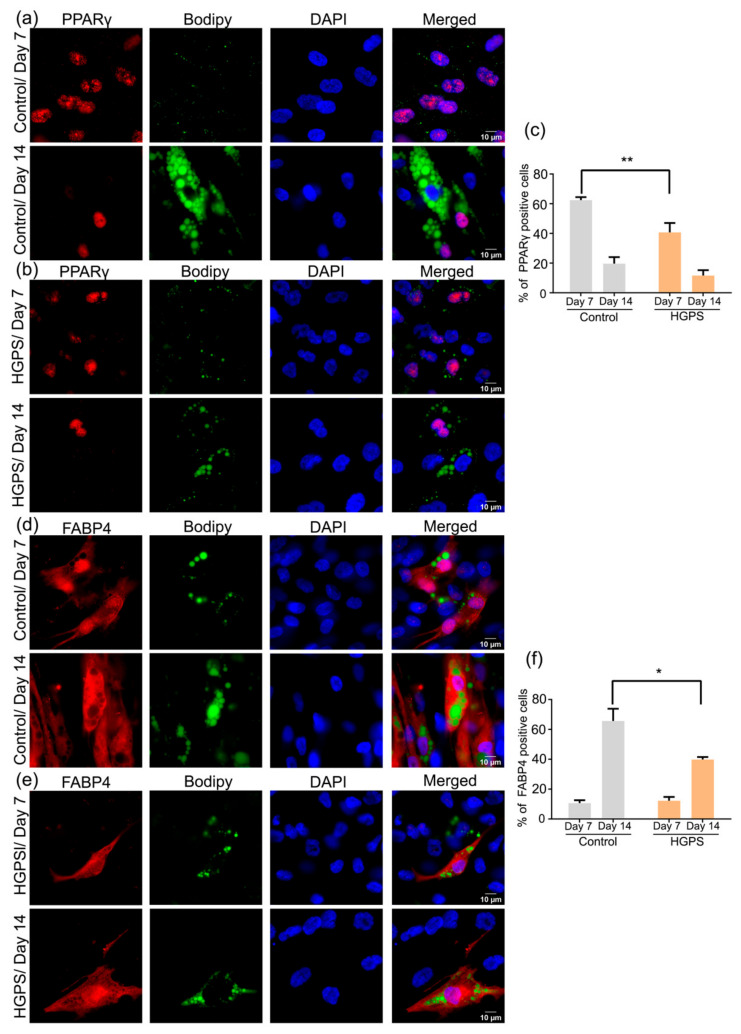
Expression of adipogenic markers in control and HGPS differentiated adipocytes. (**a**,**b**) Immunofluorescence showing the dual PPARγ and Bodipy staining in control (GMO5567A) and HGPS (HGADFN127) adipocytes at days 7 and 14 of differentiation. (**c**) Quantification of the percentage of PPARγ-positive cells at days 7 and 14 of differentiation in both control and HGPS differentiated SKPs. (**d**,**e**) FABP4 and Bodipy staining in control (GMO5567A) and HGPS (HGADFN127) differentiated adipocytes at days 7 and 14. Scale bar: 10 µm. Cells were counterstained with DAPI. Values are presented as mean ± SD (*n* = 3), * *p* < 0.05, ** *p* < 0.01, (**c**,**f**) two-way ANOVA with Tukey’s multiple comparisons test.

Having established the specificity and expression patterns of PPARγ and FABP4 in the 3T3-L1 model, we assessed the expression of these two markers in adipocyte cultures derived from control and HGPS SKPs (Figure 8). On day 7 of adipogenesis in the control and HGPS SKPs, PPARγ was expressed at high levels in both cell types, and the level of expression was reduced at day 14 of differentiation (Figure 8a,b). However, in accordance with the efficiency of control SKP differentiation, the number of PPARγ-positive cells in HGPS cultures remained lower than in control adipocyte cultures (Figure 8b). The percentage of nuclei showing positive PPARγ expression was significantly higher in the control than in the HGPS cultures at day 7 and reached similar levels in both cell types at day 14 (Figure 8c). FABP4 expression increased significantly on day 14 in differentiated SKPs derived from both cell types (Figure 8c). Nevertheless, the percentage of FABP4-positive cells was higher in control adipocyte cultures at day 14 compared with HGPS counterparts (Figure 8c). Collectively, these data indicate that HGPS SKPs can differentiate into mature adipocytes, as indicated by FABP4 expression. However, the adipogenic potential of HGPS SKPs remained lower than control SKPs.

### 3.7. Accumulation of Progerin and Increased Senescence Underlied HGPS Deffective Adipogenesis

**Figure 9:**
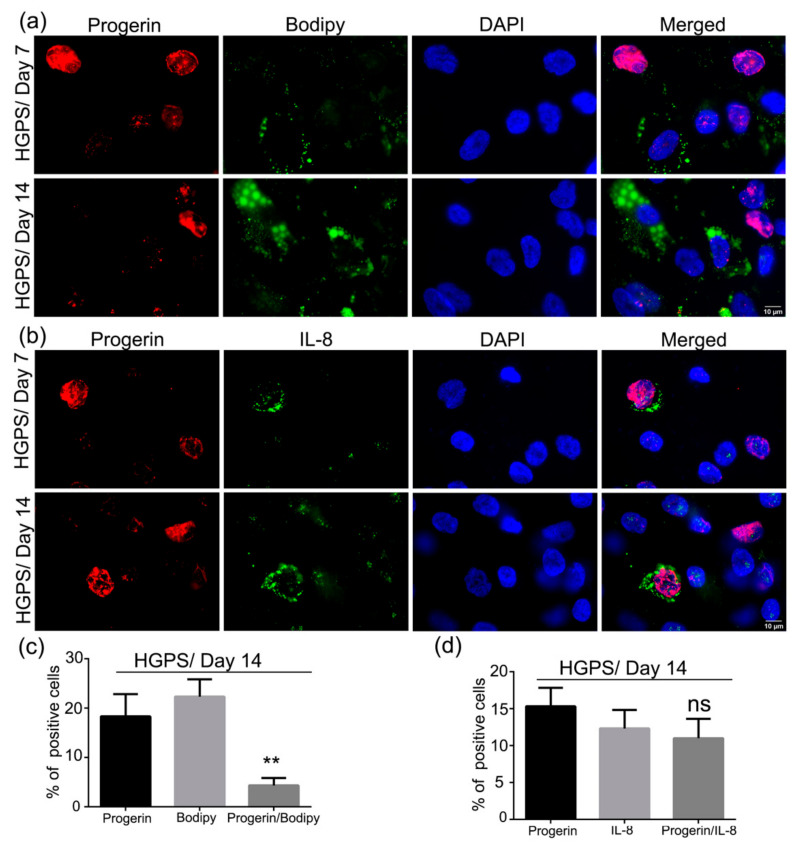
Co-expression of progerin with Bodipy and IL-8 in HGPS differentiated SKPs. (**a**) Immunofluorescence staining for progerin and Bodipy at days 7 and 14 of adipogenesis in HGPS (HGADFN127). (**b**) Immunofluorescence staining for progerin and IL-8 at days 7 and 14 of adipogenesis in HGPS. Cells were counterstained with DAPI. (**c**) Percentage of positive nuclei expressing either progerin, Bodipy, or both (progerin/Bodipy 493/503). (**d**) Percentage of progerin-positive nuclei, IL-8-positive or progerin/IL-8-positive cells. Values are presented as mean ± SD (*n* = 3), not significant (ns), ** *p* < 0.01, (**c**,**d**) unpaired *t*-test.

To determine whether senescence was responsible for the reduced number of HGPS SKPs capable of differentiating into adipocytes, we performed a series of immunohistochemical staining for progerin, IL-8, a cytokine secreted by senescent cells [45], and Bodipy. HGPS SKPs were differentiated into adipocytes and the cultures were fixed and scored for the expression of the above-mentioned proteins on days 7 and 14 (Figure 9). The nuclear accumulation of progerin has been linked to senescence in HGPS [53,54]. Cells with brightly labeled progerin-positive nuclei showed a negative cytoplasmic Bodipy signal, indicating that these cells did not differentiate into adipocytes (Figure 9a). Moreover, in the HGPS SKP adipocyte cultures, 18% of the cells were positive for progerin and 22% were positive for Bodipy (Figure 9a,c). Only 4% of the cells were positive for both progerin and Bodipy; however, in this case, the progerin signal was always weaker (Figure 9a,c).

Next, we evaluated the expression of IL-8, an established senescence marker, in relation to progerin expression (Figure 9b). Remarkably, most of the cells brightly labeled for progerin exhibited a typical cytoplasmic IL-8 signal (Figure 9b). Scoring these cultures for progerin and IL-8 signals showed that 12% of the cells were IL-8 positive and 15% were progerin-positive, among which 11% showed both signals (Figure 9d).

Altogether, these data indicate that the majority of cells brightly progerin-positive also expressed IL-8 and, consequently, were senescent. The progerin- and IL-8-positive cells did not differentiate into adipocytes, as indicated by the negative Bodipy signal. Collectively, these findings indicate that the impairment of adipogenesis in HGPS SKPs was linked to the nuclear accumulation of progerin, driving cells to premature senescence.

## 4. Discussion

In this study, we showed that the pH-SKP protocol previously described by Budel et al. [38] is effective for isolating and expanding skin-derived precursor cells from HGPS primary fibroblast cultures. A proportion of HGPS SKPs were shown to differentiate into adipocytes in the presence of ADM. The differentiated cells stained positive for both ORO and Bodipy dyes and expressed the adipogenic markers PPARγ and FABP4. The differentiation efficacy of HGPS SKPs was lower compared with control SKPs. We also showed that in both control and HGPS cultures, SKPs derived from late fibroblast passages (characterized by a high senescence index) exhibited reduced potential for differentiation into adipocytes. However, the defective adipogenesis process in HGPS SKPs was ameliorated upon treatment with baricitinib, a FDA-approved JAK1/2 inhibitor. The JAK–STAT pathway plays an important role in inflammation and is overactivated during replicative senescence [44]. This overactivation promotes the secretion of proinflammatory factors such as cytokines (IL-6, IL-8, IL-1α, and TNFα) [44]. With aging, the accumulation of senescent cells and the secretion of the SASPs drive chronic inflammation and establish an inflammatory milieu [55]. Inflammation caused by the higher abundance of senescent cells plays a critical role in inhibiting the differentiation of HGPS SKPs into adipocytes.

It remains unknown how mutations in *LMNA* can induce defects in adipocyte differentiation. Previous studies showed that HGPS-induced pluripotent stem cells (iPSCs) obtained from HGPS fibroblasts exhibited reduced adipogenic potential compared to iPSCs obtained from control fibroblasts [30]. In the cited study, progerin was suggested to interfere with late adipogenic regulators [30]. This could be attributed to the interaction of progerin with intermediate transcription factors, such as FOXQ1. FOXQ1 is suggested to be associated with the cellular dysfunction observed in the presence of lamin A mutants [56]. Other possible explanations are the physical interaction of progerin with an essential adipogenic gene, deregulation of which can activate downstream cascades that inhibit adipogenesis [30]. In contrast, prelamin A, but not lamin A, was shown to interact with SREBP1 at the nuclear envelope of pre-adipocytes [57]. SREBP1 is implicated in adipogenesis owing to its ability to trigger PPARγ expression [58]. The accumulation of prelamin A and its interaction with SREBP1 reduced the intranuclear SREBP1 levels and suppressed the activation of PPARγ, which consequently impaired adipogenesis [59].

In recent years, several therapies have been designed to improve the conditions of HGPS. These mostly target the post-translational processing of progerin, its clearance, or its downstream effects [60]. The inhibition of prelamin A farnesylation using a farnesyltransferase inhibitor (FTI) decreased progerin accumulation and reversed certain aspects of the HGPS phenotype in mouse models [61]. FTI induced weight gain in HGPS mice by increasing the adipose tissue mass [61]. Clinical trials in children with HGPS also showed weight gain post FTI treatment in some children, owing to increased muscle and bone mass, but not fat tissue [62]. Other post-translational modification blockades have also been designed, such as the inhibition of isoprenylcysteine carboxyl methyltransferase (ICMT) [60]. ICMT inhibition by lentiviral short hairpin RNAs (shICMT) prolonged the life span of *Zmpste24*^−/−^-deficient mice and increased the total body weight of the mice by increasing the adipose tissue mass [63].

Several compounds, such as rapamycin, sulforaphane, and retinoids, which activate autophagy, have been shown to enhance progerin clearance [60]. However, even though the treatment with the mTOR inhibitor rapamycin rescued the age-associated features in muscle-derived stem/progenitor cells (MDSPCs) obtained from *Zmpste24*^−/−^ mice, it suppressed the adipogenic potential of these cells [64]. Indeed, the treatment of MDSPCs during adipogenesis reduced the mRNA levels of PPARγ and LPL, which are two common adipogenic markers [64]. This is linked to the fundamental role of mTOR signaling in adipogenesis [65]. The inhibition of mTOR signaling with rapamycin also prevented SREBP-1 nuclear localization and lipogenic genes expression [66]. Similar to rapamycin, sulforaphane was shown to inhibit adipogenesis [67]. Sulforaphane inhibited adipogenic differentiation in C57BL/6N mice by inhibiting the expression of PPARγ and CCAAT/enhancer-binding protein α (C/EBPα) [67].

Other treatment strategies have been designed to attenuate the downstream toxic effects of progerin. These target ROS generation, mitochondrial dysfunction, cellular senescence, and NF-κB inhibition [60]. The NF-κB pathway was shown to be hyperactivated in both *Lmna^G609G/G609G^* and *Zmpste24*^−/−^ mouse models [68]. NF-κB activation can trigger senescence and is a key modulator of the inflammatory response and SASP secretion. Reducing chronic inflammation using sodium salicylate, an inhibitor of NF-κB signaling, increased the longevity and increased the body weight of both *Lmna^G609G/G609G^* and *Zmpste24*^−/−^ mouse models [68]. Reducing inflammation with tocilizumab, an anti-IL-6 receptor antibody, rescued the differentiation of pre-adipocytes in progeroid mice by increasing the percentage of adipogenic differentiation and the size of lipid droplets [69]. This treatment improved the terminal differentiation of adipocytes by increasing the fusion of lipid droplets [69].

In this study, we provided evidence that senescence is a negative effector of adipogenesis using the human HGPS SKPs cell model. The presence of a high number of senescent cells prior to or during adipogenic differentiation contributed to the lower abundance of mature adipocytes in the cultures. This observation was even more apparent in HGPS, as indicated by the higher incidence of senescence in adipocyte cultures derived from HGPS SKPs compared with their normal counterparts. Indeed, the senescence markers including p21, p16, and IL-8 were upregulated in HGPS SKPs and in adipocyte cultures. It is now established that an increase in the number of senescent cells is associated with the increased expression of SASPs that include proinflammatory factors [70]. SASPs can trigger a feedback loop that further increases senescence in neighboring cells, thereby creating a vicious circle that reinforces senescence entry. In this study, we showed that baricitinib treatment improved the adipogenic potential of HGPS SKPs. In a previous study, we showed that baricitinib delayed senescence by inhibiting JAK–STAT signaling and reduced the expression of proinflammatory markers in HGPS fibroblasts [44]. Along with the finding of this study, our in vitro results indicate that baricitinib treatment could promote HGPS adipogenesis by delaying senescence and inflammation. Children with HGPS show elevated levels of circulating inflammatory markers, which is indicative of chronic inflammation [71]. Our findings support the hypothesis that increased senescence and inflammation within the skin might be an underlying cause of the loss of subcutaneous fat that commences early on in patients with HGPS. However, other factors such as poor nutrition and reduced caloric intake could also play a role in the loss of adipose tissue in patients with HGPS and animal models [72]. Remarkably, *Zmpste24* knockout mice, which accumulate unfarnesylated prelamin A, exhibit only a modest reduction in adipose tissue stores in male mice [73]. This finding supports the notion that farnesylated-prelamin A and progerin impair adipogenesis [73]. Although further in vivo investigations using the HGPS mouse model are needed, we propose that baricitinib treatment could ameliorate or delay the development of generalized lipodystrophy, which is known to affect the metabolism and the appearance of children with HGPS.

## Data Availability

Data is contained within the article or Supplementary Materials.

## Supplementary Materials

The following are available online at https://www.mdpi.com/article/10.3390/cells10071598/s1, Figure S1: Senescence index of the SKPs: SA-β-gal staining on spheroids and p21 expression of original control and HGPS fibroblast cultures, Figure S2: Immunofluorescence staining for p16 and IL-8 of original control and HGPS fibroblast cultures, Figure S3: ORO staining of differentiated control and HGPS SKPs, Figure S4: 3T3-L1 differentiation and expression of adipogenic markers.
