# Supplementary material for: Impact of Progerin Expression on Adipogenesis in Hutchinson—Gilford Progeria Skin-Derived Precursor Cells
Source: Cells. 2021 Jun 25;10(7):1598. doi: 10.3390/cells10071598 (PMC8306773; doi:10.3390/cells10071598)
Supplement: Supplementary file 1 [file cells-10-01598-s001.zip › cells-1274296-supplementary.pdf]

cells MDPI

Supplementary Materials

Impact of Progerin Expression on Adipogenesis in Hutchinson-Gilford Progeria Skin-Derived Precursor Cells

Farah Najdi, Peter Krüger and Karima Djabali *

Epigenetics of Aging, Department of Dermatology and Allergy, TUM school of Medicine, Technical University of Munich (TUM), 85748 Garching, Germany; Farah.najdi@tum.de (F.N.); Peter.krueger@tum.de (P.K.)
* Correspondence: Karima Djabali, djabali@tum.de

Supplementary Figures

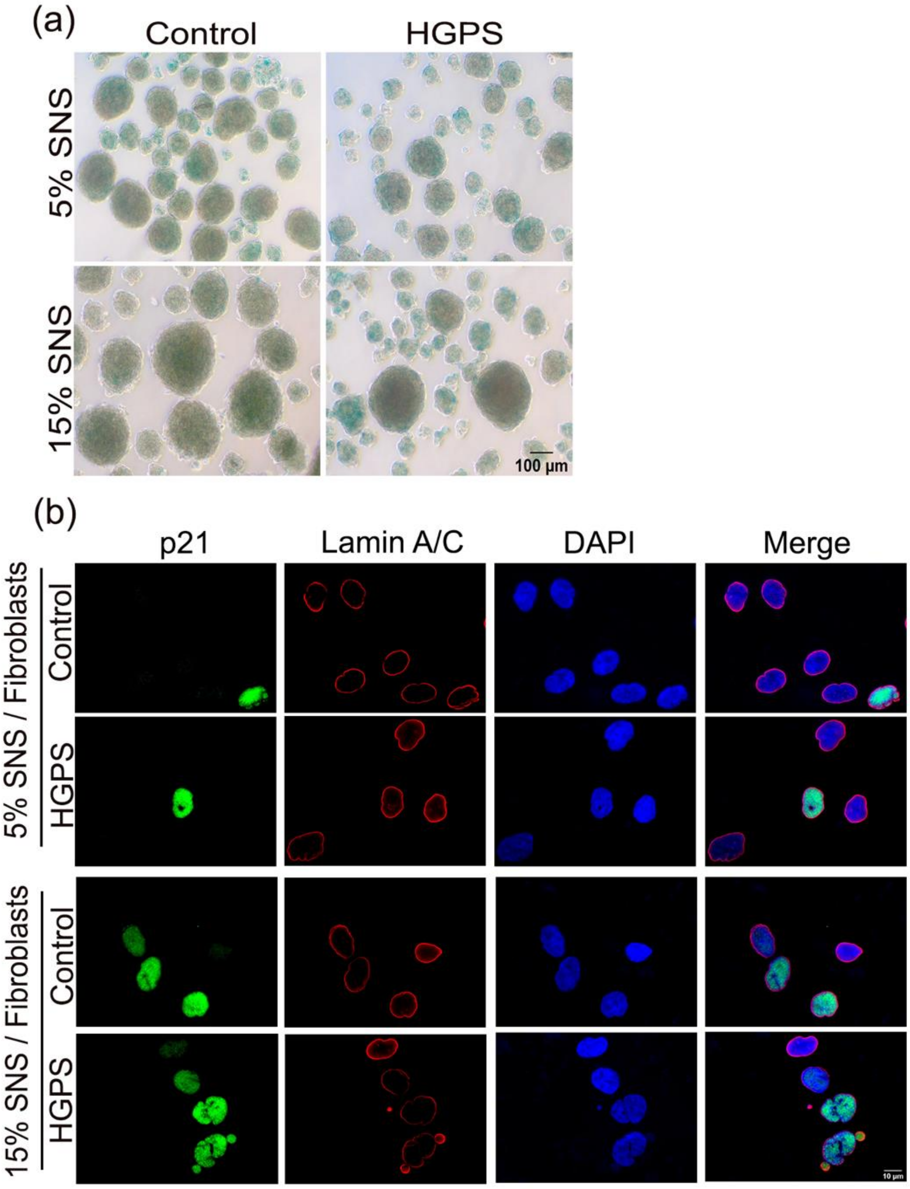

Figure S1. Senescence index of the SKPs: SA-β-gal staining of spheroids and p21 expression in original control and HGPS fibroblast cultures. (**a**) SA-β-gal test performed directly on the SKPs derived from control and HGPS SKPs starting from 5% and 15% SNS. (**b**) Immunofluorescence staining for p21 and lamin A/C in the initial control and HGPS fibroblast cultures with 5% and 15% SNS. Cells were counterstained with DAPI.

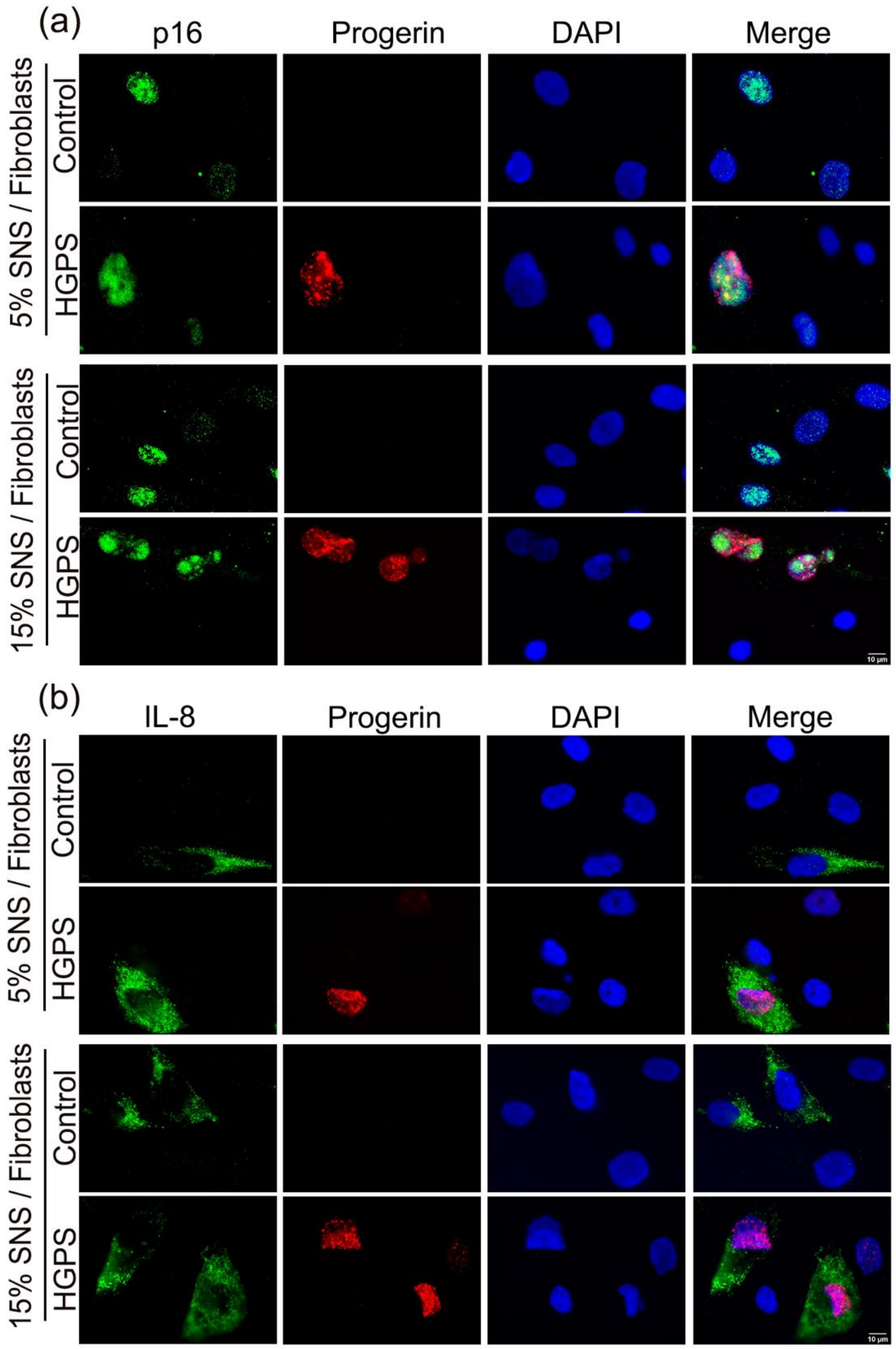

Figure S2. Immunofluorescence staining for p16 and IL-8 in original control and HGPS fibroblast cultures. (**a**) Immunofluorescence staining for p16 and progerin in initial fibroblast cultures from control and HGPS with 5% and 15% SNS. (**b**) Immunofluorescence staining for IL-8 and progerin in the initial fibroblast cultures from control and HGPS with 5% and 15% SNS. Cells were counter-stained with DAPI.

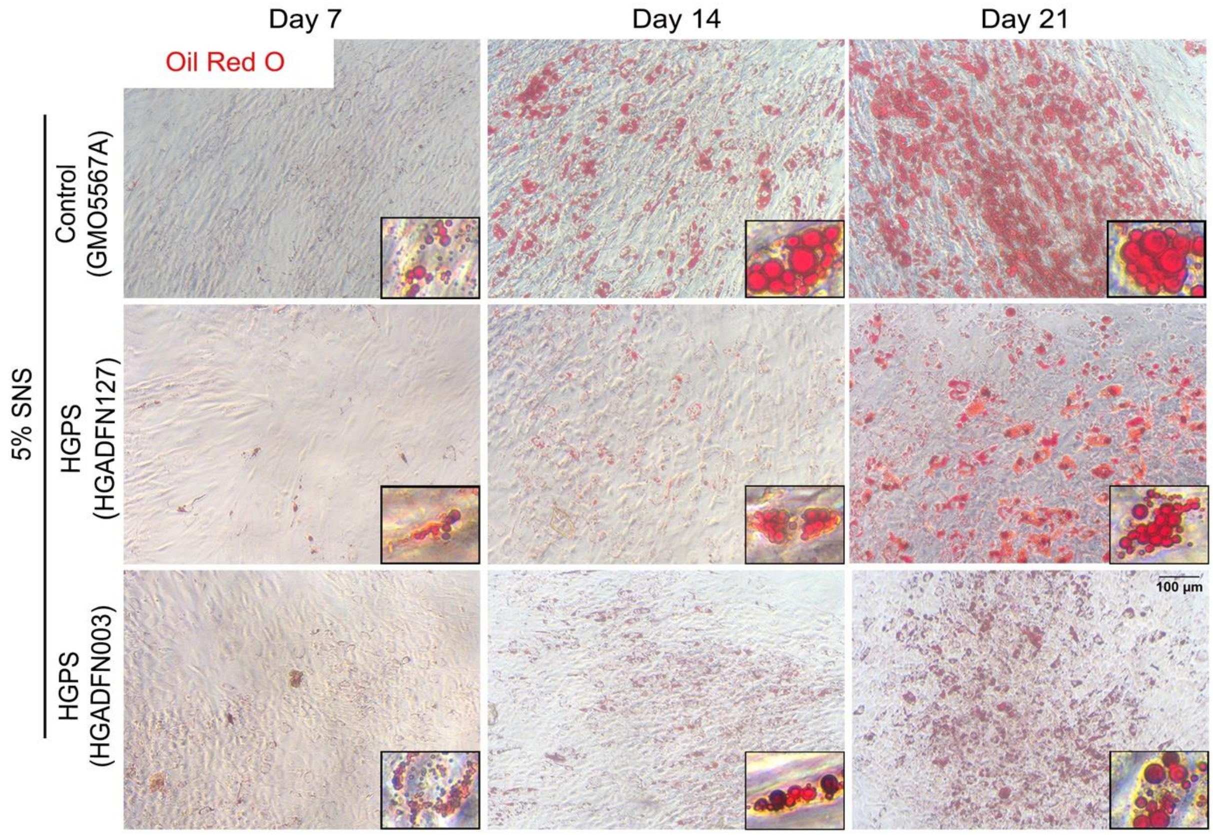

Figure S3. ORO staining of differentiated control and HGPS SKPs. ORO staining of control (GMO5567A) and HGPS (HGADFN127 and HGADFN003) differentiated adipocytes at days 7, 14 and 21 of adipogenesis, starting from 5% SNS

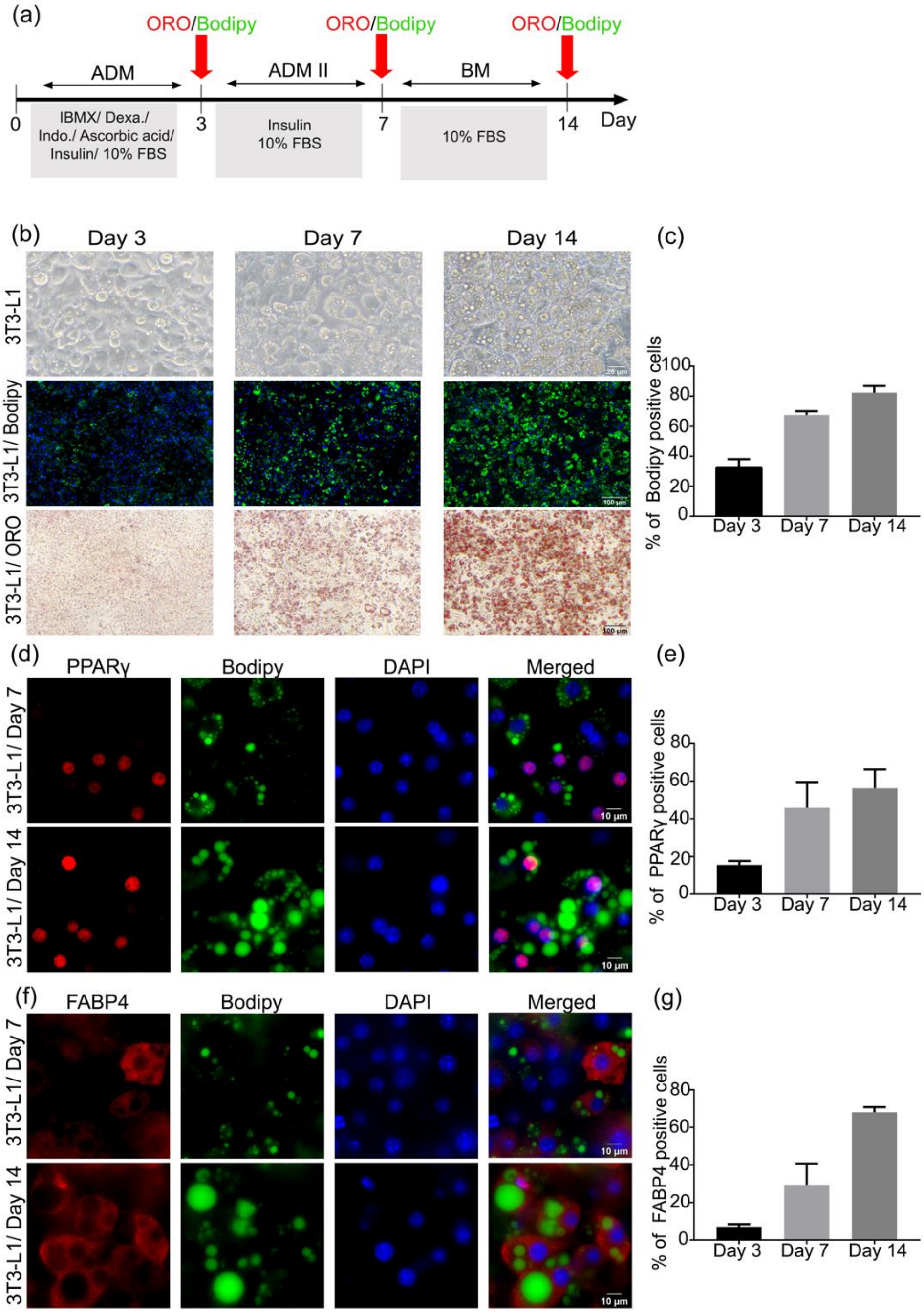

Figure S4. 3T3-L1 differentiation and expression of adipogenic markers. (**a**) Panel showing the pro-tocol for 3T3-L1 differentiation. After reaching confluence, the 3T3-L1 cells were cultured for 3 days in ADM, then replaced with ADM II containing DMEM, insulin and 10% FBS. At day 7, the media was replaced with BM containing DMEM and 10% FBS. The cells were maintained in BM till day 14 of differentiation. At days 3, day 7, and 14, the differentiated 3T3-L1 cells were fixed and stained with ORO and Bodipy. (**b**) First panel shows bright field microscopy images at days 3, 7, and 14 of 3T3-L1 adipogenic differentiation, scale bar: 25 μm. Second panel shows differentiated 3T3-L1 cells stained with Bodipy, scale bar: 100 μm. Third panel shows differentiated 3T3-L1 cells stained with ORO, scale bar: 100 μm. (**c**) Quantification of the percentage of Bodipy-positive 3T3-L1 differentiated cells. (**d**) Immunofluorescence showing dual signals for PPARγ and Bodipy in 3T3-L1 at day 7 and 14 of differentiation. (**e**) Quantification of the percentage of PPARγ positive cells at days 7 and 14 of differentiation in 3T3-L1 cells. (**f**) Immunofluorescence showing dual signals for FABP4 and Bodipy in 3T3-L1 cells on days 7 and 14 of differentiation. Cells were counter-stained with DAPI. (**g**) Quantification of percentage of FABP4 positive cells on days 7 and 14 of

adipogenic differentiation in 3T3-L1 cells. ADM: Adipocyte differentiation media, BM: Basal medium, IBMX: isobutylmethylxanthine, Dexa: dexamethasone, Indo.: indomethacine.
